# A Membrane Filter-Assisted Mammalian Cell-Based Biosensor Enabling 3D Culture and Pathogen Detection

**DOI:** 10.3390/s21093042

**Published:** 2021-04-26

**Authors:** Il-Hoon Cho, Jin-Woo Jeon, Min-Ji Choi, Hyun-Mo Cho, Jong-Sung Lee, Dong-Hyung Kim

**Affiliations:** 1 BK21 Plus Program, Department of Senior Healthcare, Graduate School, Eulji University, Daejeon 34824, Korea; ihcho@eulji.ac.kr (I.-H.C.); m1006j@g.eulji.ac.kr (M.-J.C.); 2 Department of Biomedical Laboratory Science, College of Health Science, Eulji University, 553 Sanseong-Daero, Sujeong-gu, Seongnam 13135, Korea; 3 Department of Bio-Microsystem Technology, Korea University, Seoul 02841, Korea; lunatical99@greencross.com; 4 Division of Interdisciplinary Materials Measurement Institute, Korea Research Institute of Standards and Science, Daejeon 34113, Korea; hmcho@kriss.re.kr; 5 Department of Genetic Engineering, College of Biotechnology and Bioengineering, Sungkyunkwan University, Suwon 16419, Korea

**Keywords:** cell-based biosensor, immunoassay, 3D cell culture, long-term culture, pathogen detection, membrane filter-assisted cell-based biosensor

## Abstract

We have developed a membrane filter-assisted cell-based biosensing platform by using a polyester membrane as a three-dimensional (3D) cell culture scaffold in which cells can be grown by physical attachment. The membrane was simply treated with ethanol to increase surficial hydrophobicity, inducing the stable settlement of cells via gravity. The 3D membrane scaffold was able to provide a relatively longer cell incubation time (up to 16 days) as compared to a common two-dimensional (2D) cell culture environment. For a practical application, we fabricated a cylindrical cartridge to support the scaffold membranes stacked inside the cartridge, enabling not only the maintenance of a certain volume of culture media but also the simple exchange of media in a flow-through manner. The cartridge-type cell-based analytical system was exemplified for pathogen detection by measuring the quantities of toll-like receptor 1 (TLR1) induced by applying a lysate of *P. aeruginosa* and live *E. coli*, respectively, providing a fast, convenient colorimetric TLR1 immunoassay. The color images of membranes were digitized to obtain the response signals. We expect the method to further be applied as an alternative tool to animal testing in various research areas such as cosmetic toxicity and drug efficiency.

## 1. Introduction

Mammalian cell-based biosensors utilize living cells as an analytical means of recognition to measure functional responses to biologically active analytes [1]. The biosensors usually measure morphological characteristics, electrochemical changes, gene expression levels, and metabolite concentrations of cells [1,2,3,4,5]. The cell surface receptors also provide direct immunosensing of the cellular responses induced by external stimuli [6,7,8]. Cell-based biosensing techniques have several analytical advantages, including high sensitivity, accurate selectivity, and fast reactivity [9], and can be applied in different areas such as environmental monitoring, pharmaceutical screening, and cosmetics toxicity testing [10,11,12,13].

The cell culture matrix is an important factor for improving analytical performance in cell-based biosensors. Cells immobilized onto matrices will increase the stability, sensitivity, and reproducibility of the biosensing systems [14]. Two-dimensional culture environments such as microtiter plates have been applied in various biological works; however, limited cell growth areas and culture periods for the proliferating cells over time were reported [10,11]. The contact inhibition among cells affects cell growth, which restricts the maintenance of live cells with a high density for long periods of time [15,16].

Three-dimensional cell culture methods have obtained considerable attention due to their better mimicking of in vivo situations with regard to the external stimuli using 3D tissue-like structures, i.e., scaffolds, with collagen and hydrogel [17,18,19,20]. Cell adhesion can be generated by recognition of the matrix by the cell membrane adhesion receptor [21], providing a uniform adherent layer and a high cell viability that play important roles in cell-based biosensing [9]. The 3D scaffold can provide a large surface area, as well as an effective exchange of nutrients, metabolites, and wastes for stable cell cultures for longer periods [22]. Recently, many people have extensively studied the fabrication of 3D extracellular scaffolds that provide controllable thickness and resolvable properties. These cell culture methods may allow the measurement of accumulated cellular responses against external stimuli in cell-based analyses.

A porous polyester membrane has also attracted increasing attention as a 3D solid scaffold for a cell-based biosensing platform due to its merits of low cost, simplicity, portability, disposability, and excellent biocompatibility [23]. The membrane is commonly used as a gold-conjugate release pad for a lateral-flow strip [24] which consists of different membrane pads, enabling the non-powered analysis of samples by capillary force [25]. This hydrophilic trait could also assist biomolecular interactions between antigens and antibodies, resulting in better analytical performance. Surface treatment of the membrane can induce changes in its physical and chemical properties, providing a remarkable potential for efficient 3D cell culture systems. Increasing surface hydrophobicity, e.g., through polydimethylsiloxane (PDMS) modification [26] and wax printing [27], could be one strategy for a 3D system [28].

In this work, we have developed a simple and portable type of cell-based biosensor combined with a polyester membrane filter used as a scaffold where cells can be grown by physical attachment for a long period of time. We have changed the surface property of the membrane filter to provide highly efficient cell adhesion and long-term viability. The intrinsic physical property of membrane is its high wettability, which leads to a limitation regarding the highly efficient immobilization of cells due to capillary action. Hence, we manipulated the membrane surface with ethanol to provide a hydrophobic property, enabling cell attachments to the membrane via gravity alone. For a practical application, the modified membranes were placed into a cylindrical cartridge developed using 3D printing, providing a long-term cell culture with simple cell-based analysis in a flow-through format. A human-derived lung epithelial cell line, A549, was employed for the recognition of the pathogens in this analytical system. Toll-like receptor 1 (TLR1) was employed as an indicator of bacterial inflammation induced by *P. aeruginosa* and *E. coli*. The TLR1 expression level was measured by detecting the digitized signals for the colorimetric responses. These membrane filter-based cell biosensing approaches could be available to support simple and portable platforms for point-of-care applications.

## 2. Materials and Methods

### 2.1. Materials

The epithelial lung cell line A549 (ATCC CCL-185) was supplied by American Type Culture Collection (Manassas, VA, USA). Roswell Park Memorial Institute medium (RPMI 1640) and Dulbecco’s Phosphate Buffered Saline (DPBS) with (+/+) or without (−/−) calcium and magnesium, as well as fetal bovine serum (FBS) and penicillin–streptomycin solution, were supplied by HyClone (Logan, UT, USA). The polyester membrane (PT-R5) used as a cell culture scaffold and flow-controlling pad was obtained from MDI (Gurgaon, India). The absorption pad (17CHR, chromatography grade) was provided from Whatman (Maidstone, UK). Goat anti-rabbit IgG coupled to horseradish peroxidase (HRP) was obtained from Thermo Fisher Scientific (Rockford, IL, USA). Rabbit anti-TLR1 polyclonal antibody (H-90; 0.2 mg/mL) was supplied by Santa Cruz Biotechnology (Santa Cruz, CA, USA). Casein (sodium salt type, extracted from milk), Tween-20, tryptone, yeast extract, sodium acetate, ethyl alcohol, 3,3′,5,5′-tetramethylbenzidine (TMB), Ponceau S solution, Janus Green B, and sodium chloride were purchased from Sigma (St. Louis, MO, USA). Insoluble TMB for membrane (TMBM), 0.5% trypsin–ethylenediaminetetraacetic acid (EDTA), and a 96-well cell culture plate were obtained from Moss (Pasadena, ML, USA), Gibco (Grand Island, NY, USA), and Corning Incorporated (Corning, NY, USA), respectively. Other reagents used in this study were of analytical grade.

### 2.2. Preparation of Analytical Components

*Bacterial lysate*. Bacterial lysates of *P. aeruginosa* were prepared using sonication as described elsewhere [29]. Briefly, *P. aeruginosa* was grown in Luria–Bertani broth at 37 °C for 16 h. The cells were then harvested by spinning down at 10,000× *g* at 4 °C for 20 min. The cell pellets were suspended at a concentration of 1 × 10^8^ cells/mL in 10 mM phosphate buffer (pH 7.4) containing 140 mM NaCl (PBS), and the suspension was boiled for 10 min. The heat-killed cells were lysed by ultrasound treatments for 2 min and stored on ice for 5 min. This process was repeated 5 times. The residual cell debris was removed by centrifugation at 12,000× *g* at 4 °C for 20 min. Each supernatant was measured for protein concentrations via Bradford assay [30], finally diluted to 0.1 mg/mL protein concentration (corresponding to 1 × 10^7^ cells/mL for *P. aeruginosa*) with PBS, and stored at −80 °C after being snap-frozen as aliquots.

*Live bacteria*. *Escherichia coli* (*E. coli*; ATCC 25922) was prepared in the TSB medium and incubated at 150 rpm at 37 °C for 16 h as shown in previous work [6]. The bacteria cells were quantified by the cell counting method using a hemocytometer. The cell stock (1 × 10^8^ cells/mL) was provided in TSB medium containing 50% glycerol and stored at −80 °C until use. The density of live bacteria was calculated using the colony-forming units (CFU) per mL. The cells were first spread onto TSB agar plates, and cultured at 37 °C for 24 h. The colonies were counted on the agar surface with CFU values of 200 and 1000 CFU. The concentrations were determined by multiplying the colony number by the dilution ratio.

*Mammalian cells*. The culture of mammalian cells was conducted according to the standard cell culture protocol [31]. The A549 cells were prepared in a culture medium of RPMI 1640 media supplemented with 10% FBS and 1% penicillin–streptomycin. The cells were added to a culture dish (7 mL) and cultivated in an incubator maintained at 5% CO_2_ and 37 °C until 90% cell confluency on the solid surfaces. The culture media were replaced every 2 days. To harvest the cells, the medium was removed by suction, and the cells attached to the surfaces were washed 3 times with DPBS (−/−) in the absence of calcium and magnesium. The 0.25% trypsin–EDTA solution (1 mL) was treated for 5 min followed by tapping until the cells were dispersed. The supplemented medium (20 mL) was added, and the cell suspension was then transferred into 3 different dishes (7 mL each). For stock, the cells were diluted to 3 × 10^5^ cells/mL with a culture medium containing 5% FBS and 10% dimethyl sulfoxide (DMSO), followed by quick freezing.

*Fabrication of cell culture membrane*. Circular polyester membrane (4.5 mm diameter) was prepared by the laser patterning method using the CO_2_ laser cutting tool (VLS2.30, Universal Laser Systems Inc., Scottsdale, AZ, USA). The circular membrane was first washed with de-ionized water (DIW), and immersed in 70% ethanol solution for hydrophobization, which was then dried at room temperature for 5 min. The ethanol-treated membrane was thoroughly washed by DIW 3 times and then autoclaved at 121 °C for 15 min. The membrane was dried in a dry oven maintained at 60 °C for 48 h. The leftover ethanol in the matrix was investigated by measuring the viability of immobilized cells for 48 h of cultivation using Ponceau S and Janus Green B dyes, which were treated to stain cell membrane proteins and cell organelles, respectively.

### 2.3. Analytical Procedures

*Cell attachment to 2D and 3D matrices*. The A549 suspension (3 × 10^5^ cells/mL, 75 µL) was dispensed onto a polyester membrane and microwell plate, respectively, and incubated to stably attach the cells at 5% CO_2_, 100% humidity, and 37 °C for 24 h. Culture medium was added to the 3D and 2D matrices, cultivating the cells for 24 h in the same conditions. The cells were washed with DPBS (+/+) and starved with a serum-free culture medium for 2 h.

*Cell-based immunoassay for TLR1*. The bacterial lysate was prepared from *P. aeruginosa* at 1 × 10^5^ cells/mL in the serum-free medium. The lysate was added to the cell-immobilized membrane, incubated at 37 °C for 2 h, and washed 3 times with the culture medium. The antibody specific to TLR1 (rabbit anti-TLR1) was diluted in the medium and incubated for 1 h. After washing, anti-rabbit IgG labeled with HRP was added to the membranes and further incubated for 30 min [6,32]. To detect the cellular response, HRP substrate solution (TMBM, 200 µL) was applied and kept for 15 min. The image data were obtained using a USB digital microscope (SuperEyes, Shenzhen Tak, and Assistive Technology, Shenzhen, China) and then digitized by the Java-based image processing program Image J (National Institutes of Health, Bethesda, MD, USA). For microwell analysis, soluble HRP substrate solution (200 µL) was added, and maintained for 15 min. The reaction was stopped by adding 2M sulfuric acid (50 µL), and the signal was measured at 450 nm using a microtiter plate reader (VersaMax; Molecular Devices, Sunnyvale, CA, USA). The analytical performances of the matrices were assessed regarding long-term viability (up to 16 days) and the coefficient of variation using measurements in triplicate.

## 3. Results and Discussion

### 3.1. Analytical Concept

**Figure 1:**
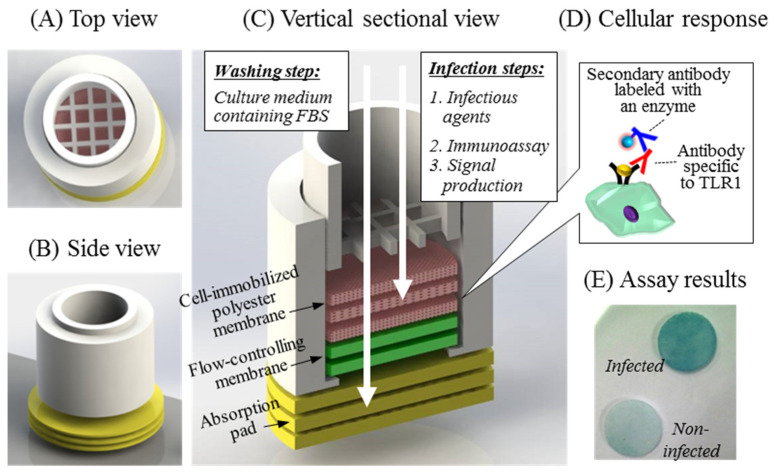
Schematics of the cell-based biosensing platform. (**A**,**B**) The design of the cell-based biosensing cartridge was fabricated by 3D printing. (**C**) The platform consisted of a sensing cartridge, cell culture scaffolds, flow-controlling membranes, and absorption pads. The flow-through format was used for culture medium change, washing, and pathogen infection in the assay. (**D**) The cellular response to the bacterial stimulation was obtained by detecting TLR1 expressed on the cell-attached membrane in the immunoassay. (**E**) The TLR1 densities were obtained by colorimetric signals on the 3D culture matrices.

Cell-based biosensors detect functional information of biologically active analytes from living cells that act as sensing elements. We designed a porous membrane filter-based cell biosensing platform forming a 3D structural scaffold as shown in Figure 1. This simple analytical platform consists of a sensing cartridge, cell culture scaffolds, flow-controlling membranes, and absorption pads (Figure 1C). The cylindrical cartridge was fabricated by 3D printing to support the scaffold membranes, not only maintaining a certain volume of culture media, but also the easy exchange of assay solutions via a flow-through format. There was a threshold at the bottom and a separate netted pressing frame at the top to stack and press the membranes (Figure 1A,B). The three polyester membranes for 3D cell culture scaffolds were able to provide a large surface area to attach the high-density cells and maintain the live cells for a long time. The flow-controlling membranes were employed to ensure a appropriate flow rate of the medium, which may have reduced cell damage from capillary force in the assay steps. Multiple absorption pads placed underneath the cartridge were applied to drain residual solution from the membranes at each assay step.

**Figure 2:**
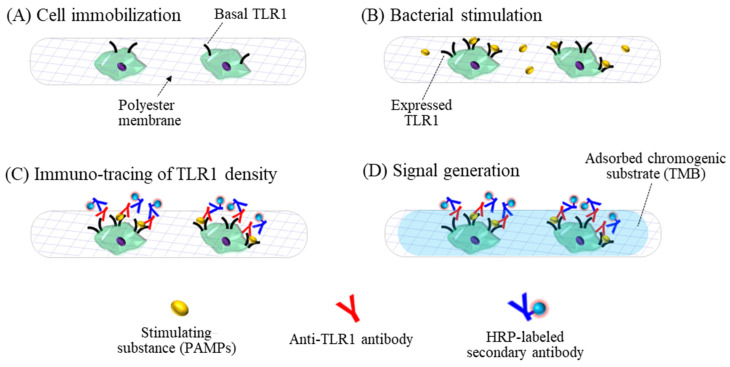
The analytical concept of the membrane filter-assisted mammalian cell-based immunoassay. (**A**) Mammalian cells were immobilized on the porous membrane treated with ethanol and (**B**) tested for the response to external stimuli using bacterial lysate and live bacteria. (**C**) The expressed TLR1 was applied as a biosensing marker for the inflammatory responses, recognized using the anti-TLR1 antibody and the enzyme-labeled secondary antibody. (**D**) The signals were generated by the chromogenic substrate absorbed by enzyme reactions on the membrane scaffold.

The analytical performance of the 3D cell-based biosensor was investigated by measuring the TLR1 on the cell membrane induced by bacterial stimulation, as shown in Figure 1D. The TLR1 response was increased depending on number of infectious bacteria in the previous study. Figure 2A–D shows the analytical design of the cell biosensing approaches using the membrane filter. We first immobilized the TLR-expressing cells onto the porous polyester membranes to detect the bacterial pathogen. The cells may be attached to the scaffold surface by a protein-mediated cell immobilization process [33]. The cellular response was induced to react sensitively to the external stimuli by a FBS-free cull culture medium lacking growth factors, halting the cell-division cycle [34]. We also applied the flow-through manner to lead to less damage to the cells during the steps of exchanging culture media and removing assay solutions. The expression levels of TLR1 were detected simply and easily using the colorimetric signals which were generated with the enzyme-labeled secondary antibody of the immunoassay (Figure 1E).

### 3.2. Fabrication of 3D Cell Culture Membrane

**Figure 3:**
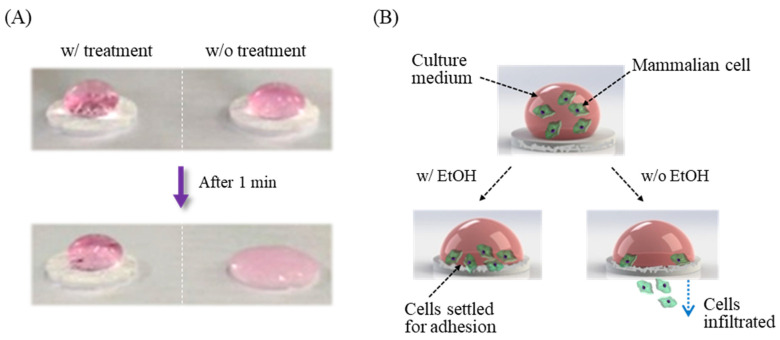
Physiochemical modification of the polyester membrane surface. (**A**) The hydrophobic surface of polyester membrane underwent ethanol treatments, maintaining the droplet form of the cell culture medium. The hydrophilic membrane without surface treatment immediately infiltrated the culture medium, reaching the porous membrane. (**B**) The number of immobilized cells was adjusted by slow infiltration of cell culture media onto the hydrophobic membrane.

The cell culture scaffolds were fabricated from the porous polyester membranes. The surface property of the instinct hydrophobic substances was modified to give hydrophilicity using different methods such as oxygen plasma treatment [35], thermal aging [36], and chemical coating [37]. The membrane support used in this study has carboxyl groups which provide a hydrophilic property in the manufacturing process. However, the hydrophilic polyester membranes infiltrate medium solutions into the inside pores very quickly by capillary action, resulting in cell loss to the outside of the culture membranes [38]. Thus, we treated ethanol for re-esterification of the membrane surfaces where carboxyl groups were functionalized as described elsewhere [39]. The esterification reaction is widely used to produce ester and water from an organic acid. Two types of the polyester membranes (one with and the other without ethanol treatment) were prepared, and a culture medium was then applied to each membrane. The ethanol-treated membrane maintained the water drop of cell suspensions without infiltrating the solution (Figure 3A; w/treatment), while the form of water drops was immediately disrupted in the absence of ethanol (Figure 3A; w/o treatment). The surface tension was created by the cohesion of water molecules, manipulating the rate of infiltration of the culture media and improving the efficiency of cell immobilization (Figure 3B). These could also be controlled not only by the area of immobilization but also by the number of immobilized cells through changes in the volume of water drops, since the suspended cells subsided over time via gravity as long as the water droplet was maintained on the surface. Cells may attach to the modified membrane scaffold by ionic interaction, with stabilization through the additional cellular process [40]. In addition, the polyester membrane has commonly been used in commercial immunodiagnostic kits as a conjugation pad that endows a 3D structure for the proper coating of biomolecules such as antibody and protein blockers, which leads to efficient reactions of the biomolecules on the membrane. We also investigated other membranes for the 3D scaffold culture system. However, for polypropylene is it is difficult to provide surface modifications with ethanol due to its resistant property to organic chemicals [41]. Polyvinyl chloride has low thermal stability, inducing the collapse of the membrane scaffold depending on the cell culture temperature [42].

**Figure 4:**
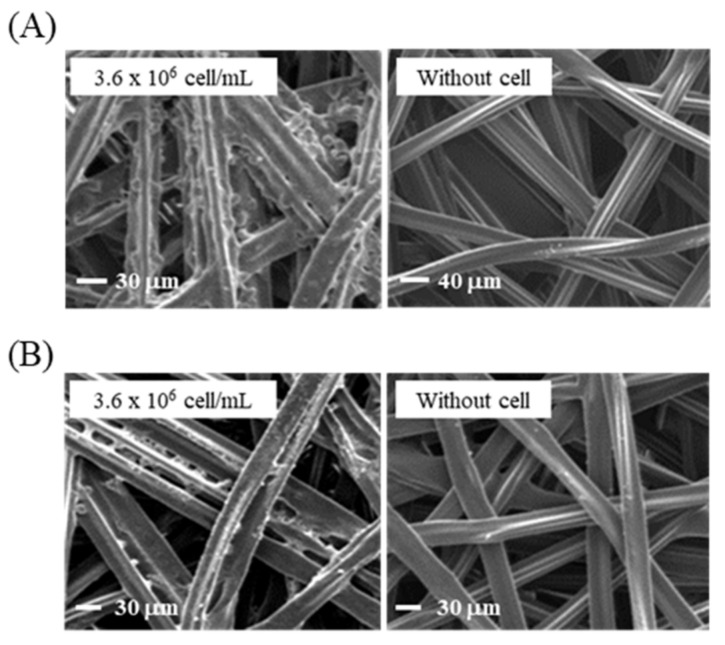
Morphological patterns of immobilized cells on the membrane scaffolds. FE-SEM images were obtained for (**A**) hydrophobic and (**B**) hydrophilic matrices, respectively, immobilized to cell concentrations of 3.6 × 10^6^ cells/mL. The cell densities of the solid matrices were compared to those of bare membranes (without cells).

The morphological patterns of immobilized cells on treated and non-treated surfaces were measured using the Hitachi 4500 (Tokyo, Japan) field emission scanning electron microscope (FE-SEM) with a standard protocol [43]. In the hydrophobic membrane treated with ethanol, cells forming certain clusters attached to polyester strands crossing each other in the form of a net could be observed (Figure 4A). However, the hydrophilic surfaces provided a relatively lower number of cells (Figure 4B). The cell densities on the two solid surfaces were clearly distinguished from the bare membrane without a cell. The pore size of >30 µm was useful for providing a 3D culture environment for the cells (10~15 µm) [44], and led to a capillary action in the cell-based immunoassay. To evaluate the cytotoxicity of ethanol-treated membranes, the viability of immobilized cells was tested using Ponceau S and Janus Green B dyes, which rapidly stained cell membrane proteins and cell organelles, respectively, for different concentrations of cells, as shown in Figure S1A–C [45]. The cells were cultured on the ethanol-treated membrane for 24 h (the upper panels), and the staining color intensities were detected on the surface where water drops were formed upon loading culture media. The intensities were increased in proportion to the cell concentrations of culture media, which also showed remarkable contrasts in color densities between the central areas and edges of the membrane surfaces. The contrasts were much clearer at 48 h of cultivation (the lower panels). The increase in staining density over culture time indicates that the immobilized cells can proliferate on membrane surfaces. This indicates that the concentration and volume of suspended cells need to be optimized to fabricate proper cell-attached membrane chips.

### 3.3. Characterization of the Cell-Based Biosensor

TLR expression from cellular responses is well known to be highly related to NF-κB activation by inflammatory reactions. The activated NF-κB induces the production of inflammatory cytokines to propagate inflammatory signals nearby, increasing expressed cell-surface receptors including TLRs to accelerate inflammatory reactions [46]. We exemplified the cell biosensing platform by detecting the colorimetric intensities of the cell-attached membranes, which reflect the expression levels of TLR1. A549 was selected as the host cell line, and offered a relatively low background in our previous work [32]. The expressed TLR1 levels of cells were measured by anti-TLR1 antibodies that were noted to be specific to the TLR1 [6], showing the interaction levels between the host cells and stimulus lysates using a secondary antibody conjugated with an enzyme.

**Figure 5:**
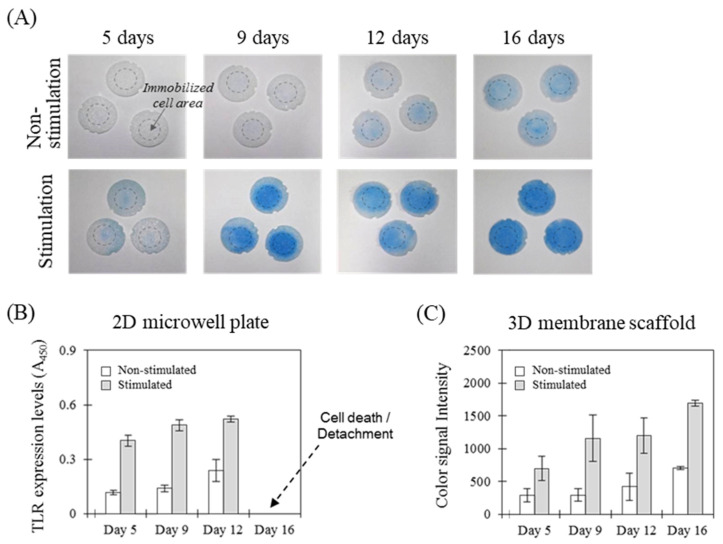
Cell-based immunoassay of TLR1 expression with different culture periods. (**A**) The cell-attached membranes were cultivated for 5, 9, 12, and 16 days, respectively, generating color images of TLR1 levels in relation to bacterial stimulation in the cell-based immunoassay. (**B**) The 2D microwell plate provided the signal digitized from the color intensities, (**C**) which was compared with that of the 3D membrane scaffold using the same format. Data are shown as the mean ± SD (*n* = 3).

The cells (3 × 10^5^ cells/mL, 75 µL) were able to actively adhere via the embedded proteins onto the membrane scaffolds, assembled to the cylindrical platform. The cell-attached membranes were first incubated at 5% CO_2_, 100% humidity, and 37 °C, and were used to detect the TLR1 receptor overexpressed by the bacterial lysate of *P. aeruginosa* of 1 × 10^5^ cells/mL over culture periods of 5, 9, 12, and 16 days, respectively, as shown in Figure 5A (stimulated). Diverse kinds of proteins included in cell culture media were adsorbed onto the solid matrices, assisting the conversion of passive adhesion (e.g., hydrophobic, coulombic, and Van der Waals forces) of cells into active adhesion [47,48]. Such adhesion is a cellular metabolic process that adjusts the shape of cells according to the surface roughness, which more vigorously expands the area of cell proliferation from the center to the edges of the membrane surface. The negative control was provided through the membrane without external stimuli and was used to subtract non-specific signals from the TLR1 levels using the same format (Figure 5A, non-stimulated). The non-specific responses were gradually increased by the TLR1 induced from intercellular stimulation with the high cell density of culture matrices in the absence of external stimulation.

Figure 5B showed the quantitative intensities for TLR expression levels on the 2D plate. The 2D cell matrices provided greater expression levels of TLR1 over a cell culture period of 5 days, showing a coefficient of variation of <7.4%. Although the 3D scaffold provided a higher coefficient of variation of <30.4%, the cellular response increased in proportion to the culture period and was clearly higher than that of the negative control. The membrane was also able to provide a relatively longer cell culture time (16 days) as compared to the 2D plastic plate (Figure 5C). The merit of a long culture period is that it can induce an enhanced analytical performance of TLR receptor-based immunoanalysis by sensitively detecting the accumulated cellular response to the pathogenic bacterium, supporting the cyclic biosensing of TLR regulation in inflammation inhibition models which can consistently test anti-inflammatory substance effects over long periods of time [32].

**Figure 6:**
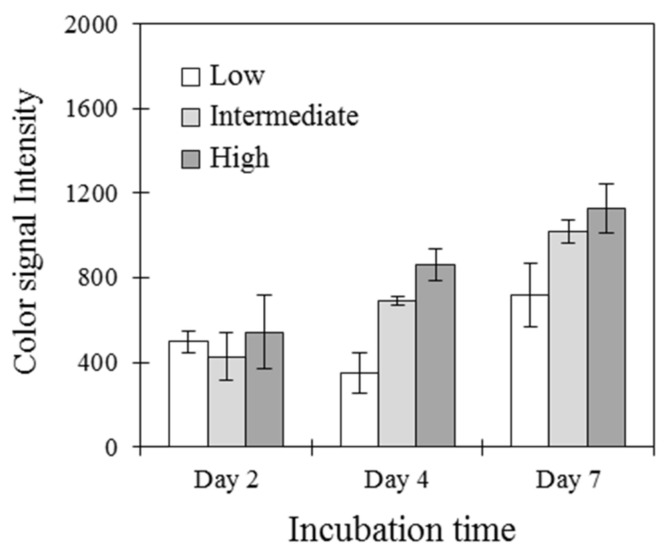
Comparison of TLR1 expression levels with different cell densities. Cells at low, intermediate, and high concentrations were provided to the membrane filter-assisted biosensing cartridge. The color signals were detected for TLR1 levels overexpressed from the bacterial lysate at different incubation times of 2, 4, and 7 days from the initial culture.

We determined the initial cell density for an appropriate time period and for high performance of the assay. Three different densities of cells were prepared in the same culture media, added for the attachment of cells on the membrane scaffolds, and then the immobilized cells were cultivated for pre-determined culture periods (days 2, 4, and 7). The inflammatory reactions were induced by the bacterial lysate on the cell-attached membrane, with subsequent evaluation using immunoassays to detect TLR1, as shown in Figure 6. The initial concentration of immobilized cells was highly relevant to the starting point of cellular response to stimuli. The low density of 1 × 10^6^ cells/mL delayed the starting point of the cell-based immunoassay, while the response signals were excellently distinguished on the membrane scaffolds cultured for 4 days with intermediate (3 × 10^6^ cells/mL) and high (6 × 10^6^ cells/mL) concentrations, providing high-performance cell-based analysis.

**Figure 7:**
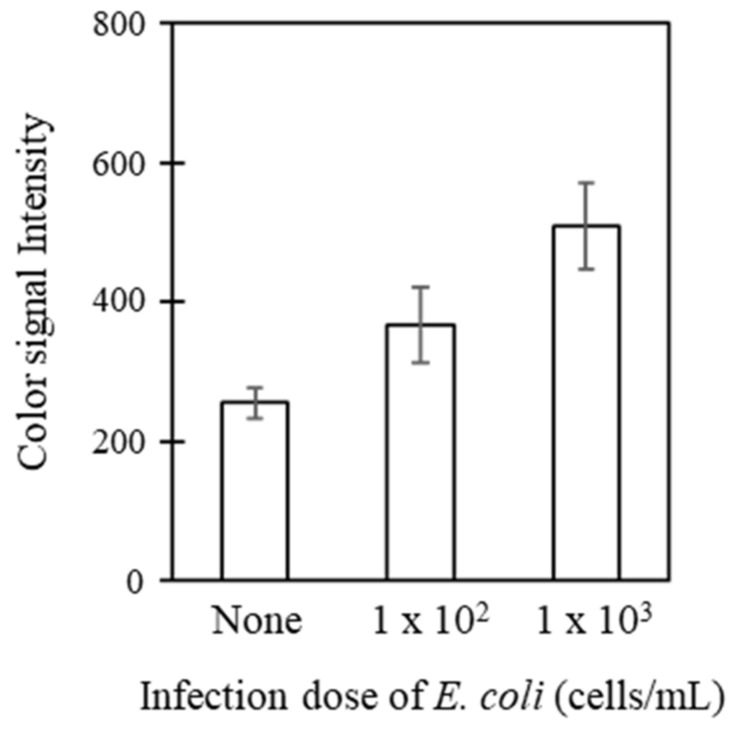
Analytical evaluation of the system with respect to infection by live bacteria. Host cells at 3 × 10^6^ cells/mL were prepared on the membrane scaffolds and incubated for 4 days. Different concentrations of *E. coli* (0, 1 × 10^2^ and 1 × 10^3^ cells/mL) were inoculated and analyzed as described elsewhere.

We further applied the biosensor system for the detection of live bacteria. *E. coli* was inoculated at different concentrations (0, 1 × 10^2^, and 1 × 10^3^ cells/mL), and was then analyzed by measuring TLR1. Figure 7 shows the detection capability of the system with regard to *E. coli* infection, with excellent analytical performance through detection at as low as 1 × 10^2^ cells/mL. This analytical evaluation indicates that the system can be extended to use for the real-time monitoring of bacterial infections.

The membrane filter-assisted mammalian cell-based assay could thus be achieved using this cartridge system, which supported maintained cellular activity, shortened assay times, and long-term cultures. The 3D culture matrices were able to provide maintained activity for a long time by easily inducing intercellular signaling. The assay time was also shortened as compared to 2D matrices through enhancement of the antigen–antibody reaction in the high-cell-density per-unit area. The flow-through format was useful for changing assay media and removing residual solutions without cell damage during the assay steps. Bacteria detection was simple and rapid using the plastic cartridge with the cell-attached membrane matrices, and as such, non-experts would easily be able to conduct cell-based analyses.

## 4. Conclusions

A polyester membrane can be utilized as a solid scaffold for long-term cell culture by surface modification using ethanol. The membrane provides a large surface area for cell immobilization and improves the diffusion rate of assay solutions, which is beneficial for long-term cell cultures and rapid assays. This cartridge-type biosensing kit could be applied in various research areas such as environmental toxicity, water quality monitoring, and drug screening. Although there are other natural (collagen, gelatin, fibrinogen, alginate) and synthetic (PEG, PMMA, polystyrene) scaffolds, the 3D culture system introduced here can easily be constructed solely via cell incubation using the membrane filter-based support structure, without additional processes. Furthermore, the cylindrical cartridge helps to simply provide 3D environments by only stacking the membranes, which may lead to enhanced intercellular communication. We expect that this membrane filter-based cell biosensing method will be available to point-of-care platforms in combination with lateral-flow immunochromatography kits as an alternative tool to animal testing in various research areas such as cosmetic toxicity and drug efficiency. It only requires cheap cell chips (below one US dollar), and has increased reproducibility in terms of analytical performance, encouraging the application of cell biosensing approaches in commercial fields. Currently, these systems are being applied to further studies involving the fabrication of skin cell-based 3D cell culture platforms using keratinocytes (HaCaT) and fibroblasts (Hs68), enabling the simultaneous detection of inflammatory cytokines (IL-6, IL-8, and TNF-α) under external physical and chemical stimuli such as ultraviolet (UV) light, lipopolysaccharide, and nickel (II) sulfate (NiSO_4_).

## Data Availability

This study did not use any reported data.

## Supplementary Materials

The following are available online at https://www.mdpi.com/article/10.3390/s21093042/s1, Figure S1: Dye-based analysis of cell viability.

## References

[1] Banerjee P., Bhunia A.K. (2009). Mammalian cell-based biosensors for pathogens and toxins. Trends Biotechnol..

[2] Wang P., Xu G., Qin L., Xu Y., Li Y., Li R. (2005). Cell-based biosensors and its application in biomedicine. Sens. Actuators B Chem..

[3] Liu Q., Wu C., Cai H., Hu N., Zhou J., Wang P. (2014). Cell-Based Biosensors and Their Application in Biomedicine. Chem. Rev..

[4] Bawolak M.-T., Gera L., Morissette G., Bouthillier J., Stewart J.M., Gobeil L.-A., Lodge R., Adam A., Marceau F. (2009). Fluorescent Ligands of the Bradykinin B1 Receptors: Pharmacologic Characterization and Application to the Study of Agonist-Induced Receptor Translocation and Cell Surface Receptor Expression. J. Pharmacol. Exp. Ther..

[5] Ebert M.S., Neilson J.R., Sharp P.A. (2007). MicroRNA sponges: Competitive inhibitors of small RNAs in mammalian cells. Nat. Methods.

[6] Cho I.-H., Jeon J.-W., Paek S.-H., Kim D.-H., Shin H.-S., Ha U.-H., Seo S.-K., Paek S.-H. (2012). Toll-Like Receptor-Based Immuno-Analysis of Pathogenic Microorganisms. Anal. Chem..

[7] Wolf M., Zimmermann M., Delamarche E., Hunziker P. (2007). Screening cell surface receptors using micromosaic immunoassays. Biomed. Microdevices.

[8] Kohl T.O., Ascoli C.A. (2017). Direct and Indirect Cell-Based Enzyme-Linked Immunosorbent Assay. Cold Spring Harb. Protoc..

[9] Lu X., Ye Y., Zhang Y., Sun X. (2020). Current research progress of mammalian cell-based biosensors on the detection of foodborne pathogens and toxins. Crit. Rev. Food Sci. Nutr..

[10] Hertzberg R.P., Pope A.J. (2000). High-throughput screening: New technology for the 21st century. Curr. Opin. Chem. Biol..

[11] Slaughter G.E., Hobson R. (2009). An impedimetric biosensor based on PC 12 cells for the monitoring of exogenous agents. Biosens. Bioelectron..

[12] Kloss D., Fischer M., Rothermel A., Simon J.C., Robitzki A.A. (2008). Drug testing on 3D in vitro tissues trapped on a microcavity chip. Lab Chip.

[13] Xu G., Ye X., Qin L., Xu Y., Li Y., Li R., Wang P. (2005). Cell-based biosensors based on light-addressable potentiometric sensors for single cell monitoring. Biosens. Bioelectron..

[14] Gao G., Qian J., Fang D., Yu Y., Zhi J. (2016). Development of a mediated whole cell-based electrochemical biosensor for joint toxicity assessment of multi-pollutants using a mixed microbial consortium. Anal. Chim. Acta.

[15] Pollack R.E., Green H., Todaro G.J. (1968). Growth control in cultured cells: Selection of sublines with increased sensitivity to contact inhibition and decreased tumor-producing ability. Proc. Natl. Acad. Sci. USA.

[16] Todaro G.J., Lazar G.K., Green H. (1965). The initiation of cell division in a contact-inhibited mammalian cell line. J. Cell. Comp. Physiol..

[17] Grinnell F. (2003). Fibroblast biology in three-dimensional collagen matrices. Trends Cell Biol..

[18] Grinnell F., Ho C.-H., Tamariz E., Lee D.J., Skuta G. (2003). Dendritic Fibroblasts in Three-dimensional Collagen Matrices. Mol. Biol. Cell.

[19] Khetan S., Burdick J.A. (2010). Patterning network structure to spatially control cellular remodeling and stem cell fate within 3-dimensional hydrogels. Biomaterials.

[20] Lee S.-H., Moon J.J., West J.L. (2008). Three-dimensional micropatterning of bioactive hydrogels via two-photon laser scanning photolithography for guided 3D cell migration. Biomaterials.

[21] Huxley-Jones J., Robertson D.L., Boot-Handford R.P. (2007). On the origins of the extracellular matrix in vertebrates. Matrix Biol..

[22] Baker B.M., Chen C.S. (2012). Deconstructing the third dimension—how 3D culture microenvironments alter cellular cues. J. Cell Sci..

[23] Rahman M. (2012). Degradation of Polyesters in Medical Applications.

[24] Khlebtsov B.N., Tumskiy R.S., Burov A.M., Pylaev T.E., Khlebtsov N.G. (2019). Quantifying the Numbers of Gold Nanoparticles in the Test Zone of Lateral Flow Immunoassay Strips. ACS Appl. Nano Mater..

[25] Koczula K.M., Gallotta A. (2016). Lateral flow assays. Essays Biochem..

[26] Moghadas H., Saidi M.S., Kashaninejad N., Nguyen N.-T. (2018). A high-performance polydimethylsiloxane electrospun membrane for cell culture in lab-on-a-chip. Biomicrofluidics.

[27] Jiang D., Ge P., Wang L., Jiang H., Yang M., Yuan L., Ge Q., Fang W., Ju X. (2019). A novel electrochemical mast cell-based paper biosensor for the rapid detection of milk allergen casein. Biosens. Bioelectron..

[28] Ng K., Gao B., Yong K.W., Li Y., Shi M., Zhao X., Li Z., Zhang X., Pingguan-Murphy B., Yang H. (2017). Paper-based cell culture platform and its emerging biomedical applications. Mater. Today.

[29] Shin H.S., Ha U.H. (2011). Up-regulation of bradykinin B2 receptor by Pseudomonas aeruginosa via the NF-κB pathway. Curr. Microbiol..

[30] Zor T., Selinger Z. (1996). Linearization of the Bradford Protein Assay Increases Its Sensitivity: Theoretical and Experimental Studies. Anal. Biochem..

[31] Giard D.J., Aaronson S.A., Todaro G.J., Arnstein P., Kersey J.H., Dosik H., Parks W.P. (1973). In Vitro Cultivation of Human Tumors: Establishment of Cell Lines Derived From a Series of Solid Tumors2. J. Natl. Cancer Inst..

[32] Jeon J.-W., Ha U.-H., Paek S.-H. (2014). In Vitro Inflammation Inhibition Model Based on Semi-Continuous Toll-Like Receptor Biosensing. PLoS ONE.

[33] Assoian R.K. (1997). Anchorage-dependent Cell Cycle Progression. J. Cell Biol..

[34] Chen M., Huang J., Yang X., Liu B., Zhang W., Huang L., Deng F., Ma J., Bai Y., Lu R. (2012). Serum Starvation Induced Cell Cycle Synchronization Facilitates Human Somatic Cells Reprogramming. PLoS ONE.

[35] Tan S.H., Nguyen N.-T., Chua Y.C., Kang T.G. (2010). Oxygen plasma treatment for reducing hydrophobicity of a sealed polydimethylsiloxane microchannel. Biomicrofluidics.

[36] Eddington D.T., Puccinelli J.P., Beebe D.J. (2006). Thermal aging and reduced hydrophobic recovery of polydimethylsiloxane. Sens. Actuators B Chem..

[37] Xiao D., Zhang H., Wirth M. (2002). Chemical Modification of the Surface of Poly(dimethylsiloxane) by Atom-Transfer Radical Polymerization of Acrylamide. Langmuir.

[38] Vroman L. (1962). Effect of absorbed proteins on the wettability of hydrophilic and hydrophobic solids. Nature.

[39] Yadav G.D., Thathagar M.B. (2002). Esterification of maleic acid with ethanol over cation-exchange resin catalysts. React. Funct. Polym..

[40] Ito Y. (1999). Surface micropatterning to regulate cell functions. Biomaterials.

[41] Da Cunha C.B., Lopes P.P., Mayer F.D., Hoffmann R. (2018). Assessment of Chemical and Mechanical Properties of Polymers Aiming to Replace the Stainless Steel in Distillation Column. Mater. Res..

[42] Marongiu A., Faravelli T., Bozzano G., Dente M., Ranzi E. (2003). Thermal degradation of poly(vinyl chloride). J. Anal. Appl. Pyrolysis.

[43] Seo S.-M., Kim S.-W., Park J.-N., Cho J.-H., Kim H.-S., Paek S.-H. (2016). A fluorescent immunosensor for high-sensitivity cardiac troponin I using a spatially-controlled polymeric, nano-scale tracer to prevent quenching. Biosens. Bioelectron..

[44] Jiang R.-D., Shen H., Piao Y.-J. (2010). The morphometrical analysis on the ultrastructure of A549 cells. Rom. J. Morphol. Embryol..

[45] Raspotnig G., Fauler G., Jantscher A., Windischhofer W., Schachl K., Leis H.J. (1999). Colorimetric Determination of Cell Numbers by Janus Green Staining. Anal. Biochem..

[46] Hennessy E.J., Parker A.E., O’Neill L.A.J. (2010). Targeting Toll-like receptors: Emerging therapeutics?. Nat. Rev. Drug Discov..

[47] Okano T., Yamada N., Okuhara M., Sakai H., Sakurai Y. (1995). Mechanism of cell detachment from temperature-modulated, hydrophilic-hydrophobic polymer surfaces. Biomaterials.

[48] Weiss L., Blumenson L.E. (1967). Dynamic adhesion and separation of cellsin vitro II. Interactions of cells with hydrophilic and hydrophobic surfaces. J. Cell. Physiol..

