# Supplementary material for: A Membrane Filter-Assisted Mammalian Cell-Based Biosensor Enabling 3D Culture and Pathogen Detection
Source: Sensors (Basel). 2021 Apr 26;21(9):3042. doi: 10.3390/s21093042 (PMC8123675; doi:10.3390/s21093042)
Supplement: Supplementary file 1 [file sensors-21-03042-s001.zip › sensors-1167074-supplementary.pdf]

Membrane Filter-Assisted Mammalian Cell-Based Biosensor Enabling 3D Culture and Pathogen Detection

Il-Hoon Cho[1,5,a], Jin-Woo Jeon[2,a], MinJi Choi[1], Hyun Mo Cho[3], Jongsung Lee[4,*] and Dong Hyung Kim[3,]**

[1]BK21 Plus Program, Department of Senior Healthcare, Graduate School, Eulji University, Daejeon 34824, Republic of Korea

[2]Department of Bio-Microsystem Technology, Korea University, 145 Anam-Ro, Seongbuk-Gu, Seoul 02841, Republic of Korea

[3]Division of Interdisciplinary Materials Measurement Institute, Korea Research Institute of Standards and Science, 267 Gajeong-Ro, Yuseong-Gu, Daejeon 34113, Republic of Korea

[4]Department of Genetic Engineering, College of Biotechnology and Bioengineering, Sungkyunkwan University, Suwon City, 164-19 Gyeonggi-Do, Republic of Korea

[5]Department of Biomedical Laboratory Science, College of Health Science, Eulji University, 553 Sanseong-Daero, Sujeong-Gu, Seongnam, Gyeonggi-Do 13135, Republic of Korea

Running head: *Membrane filter-assisted cell-based biosensor*

[a] These authors equally contributed to this work

*,** These are corresponding authors

E-mail: donghyung.kim@kriss.re.kr

Tel: +82-42-868-5819, Fax: +82-42-868-5843

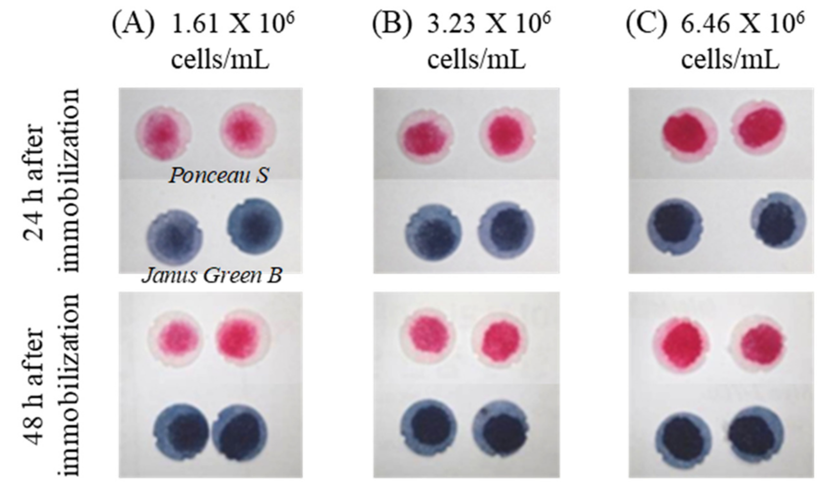

Figure S1. Dye-based analysis of cell viability. The loading cells with different concentrations were cultured for 24 h and 48 h, respectively (A to C). The cell viability was tested using Ponceau S and Janus Green B dyes on the membrane matrices.
